# Toll-Like Receptor 9-Activation during Onset of Myocardial Ischemia Does Not Influence Infarct Extension

**DOI:** 10.1371/journal.pone.0104407

**Published:** 2014-08-15

**Authors:** Ingrid Kristine Ohm, Erhe Gao, Maria Belland Olsen, Katrine Alfsnes, Marte Bliksøen, Jonas Øgaard, Trine Ranheim, Ståle Haugset Nymo, Yangchen Dhondup Holmen, Pål Aukrust, Arne Yndestad, Leif Erik Vinge

**Affiliations:** 1 Research Institute of Internal Medicine, Oslo University Hospital Rikshospitalet, Oslo, Norway; 2 Faculty of Medicine, University of Oslo, Oslo, Norway; 3 Center for Heart Failure Research, University of Oslo, Oslo, Norway; 4 Center for Translational Medicine, School of Medicine, Temple University, Philadelphia, Pennsylvania, United States of America; 5 Section of Clinical Immunology and Infectious Diseases, Oslo University Hospital Rikshospitalet, Oslo, Norway; 6 K.G. Jebsen Inflammatory Research Center, University of Oslo, Oslo, Norway; 7 Department of Cardiology, Oslo University Hospital Rikshospitalet, Oslo, Norway; 8 K.G. Jebsen Cardiac Research Center, University of Oslo, Oslo, Norway; University Hospital Medical Centre, Germany

## Abstract

**Aim:**

Myocardial infarction (MI) remains a major cause of death and disability worldwide, despite available reperfusion therapies. Inflammatory signaling is considered nodal in defining final infarct size. Activation of the innate immune receptor toll-like receptors (TLR) 9 prior to ischemia and reperfusion (I/R) reduces infarct size, but the consequence of TLR9 activation timed to the onset of ischemia is not known.

**Methods and Results:**

The TLR9-agonist; CpG B was injected i.p. in C57BL/6 mice immediately after induction of ischemia (30 minutes). Final infarct size, as well as area-at-risk, was measured after 24 hours of reperfusion. CpG B injection resulted in a significant increase in circulating granulocytes and monocytes both in sham and I/R mice. Paradoxically, clear evidence of reduced cardiac infiltration of both monocytes and granulocytes could be demonstrated in I/R mice treated with CpG B (immunocytochemistry, myeloperoxidase activity and mRNA expression patterns). In addition, systemic TLR9 activation elicited significant alterations of cardiac inflammatory genes. Despite these biochemical and cellular changes, there was no difference in infarct size between vehicle and CpG B treated I/R mice.

**Conclusion:**

Systemic TLR9-stimulation upon onset of ischemia and subsequent reperfusion does not alter final infarct size despite causing clear alterations of both systemic and cardiac inflammatory parameters. Our results question the clinical usefulness of TLR9 activation during cardiac I/R.

## Introduction

Despite great advances in treatment strategies over the last years, myocardial infarction (MI) remains a major cause of death and disability worldwide. To reduce myocardial damage and improve clinical outcome, restoration of blood flow to the heart, either by thrombolytic therapy or percutaneous coronary intervention (PCI), is vital. Paradoxically, the process of reperfusion itself greatly contributes to myocardial injury, and has been suggested to account for up to 50% of the final infarct size [1], [2]. Significant improvements in future treatments of MI are therefore likely to combine current therapy and targeting of molecular pathways involved in ischemia/reperfusion (I/R) injuries.

The mechanisms involved in I/R injury are complex and not yet fully understood [1], [2]. The changes that occur upon ischemia followed by reperfusion involve an array of biochemical and metabolic changes that mediate detrimental effects within the myocardium [1], [2]. These changes include mitochondrial re-energization, generation of reactive oxygen species (ROS), intracellular Ca^2+^-overload and rapid restoration of physiological pH; all of which act in concert and cause opening of mitochondrial permeability transitioning pore and subsequent cellular death [1], [2]. A consequence of I/R injury is activation of innate and subsequent adaptive immune responses which is important for adequate healing following MI [3]. However, strong evidence points to detrimental consequences if such activity is unbalanced or sustained [3].

Within the innate immune system, pattern recognition receptors (PRRs) recognize several endogenous proteins, lipids, and nucleic acids that act as damage signals (collectively called damage associated molecular patterns; DAMPs) when released upon cellular stress or injury such as during MI [4]–[6]. Toll-like receptors (TLRs) constitute one of the largest subfamilies of PRRs [7]. Of these, TLR9 has been demonstrated to specifically detect unmethylated DNA, rich in cytosine-phosphate-guanine (CpG) motifs [8]. Recent work has identified mitochondrial DNA to function as a DAMP, causing activation of the innate immune system through TLR9 [9], [10]. On this notion, our group have previously reported increased circulating levels of mtDNA upon PCI of human MI [11].

The consequence of TLR signaling in I/R remains unclear. Activation of TLR9 (and TLR2 and TLR4) *prior* to I/R lead to reduced infarct extension and improved cardiac function [5], [12]–[15]. From a clinical point of view, an important and hereto unaddressed question is whether activation of said receptors *during onset* of I/R also impacts subsequent myocardial damage. Thus, in the current study we investigated the pathophysiological consequence of intervening with the TLR9-agonist CpG B during onset of ischemia.

## Methods

### Ethics statement

All animal experiments were approved by the Norwegian Animal Research Committee and were in accordance with the “Principle of laboratory animal care” (NIH publication No. 86-23, revised 1985).

### Minimally invasive myocardial *in vivo* ischemia/reperfusion

The procedure of surgical induction of I/R has in detail been described previously [16]. Briefly, male and female C57BL/6 mice (8 weeks) were anesthetized using a mixture of 2% isoflurane gas and 98% oxygen, hearts were exteriorized through the forth intercostal space and subsequently the left anterior descending coronary artery was ligated using a slipknot. Mice were then immediately injected i.p. 100 µl of a TLR9-agonist (CpG B, 50 µg, ODN 1668 class B, Invivogen, San Diego, CA, USA) or vehicle (PBS). Mice were returned to a stringent temperature -and humidity controlled cabinet for 30 minutes before the slipknot was released and the myocardium subsequently reperfused. Analgesia was provided through i.p. injection of buprenorphine (0.1 mg/kg), immediately and 12 hours after operation. Mice were then kept for 3 or 24 hours after reperfusion, before re-anesthetized and subsequent euthanization by extracting the hearts. Processing of hearts and blood were performed as described below.

### *In vivo* pharmacological assessments of CpG B efficiency

To validate the cardiac bioavailability of the TLR9-agonist CpG B within a relevant time-frame, mice (n = 5 for each time-point) were i.p. injected 50 µg/100 µl CpG B and hearts were extracted at different time points (10 min, 30 min, 1 h, 3 h). PBS injected mice euthanized after 10 min and 3 h (n = 5 at both time-points) served as controls. RNA was extracted and cDNA was synthesized as described below. Based upon previous experience, we chose cardiac mRNA expression of TNFα and CXCL2 as readout for TLR9-stimulated transcriptional activity.

### Determination of infarct size

Evaluation of infarct size was performed as previously described [17]. Briefly, after 24 hours of reperfusion, mice were anesthetized, the thoracic cavity opened and the slipknot re-ligated. The aorta ascendens was subsequently clamped and approximately 0.2 ml 2% Evans blue dye was injected into the proximal portion of the aorta, this allowing coronary perfusion and visualization of the area at risk (AaR). Hearts were subsequently excised, let to freeze on dry-ice before cross-sectioned into standardized 1 mm slices by the use of a mouse heart slicer matrix (Zivic Instruments, Pittsburgh, PA, USA). The slices were further incubated for 15 min in 1% 2,3,5-triphenyltetrazolium chloride (TTC, Sigma Aldrich, St. Louis, MO, USA) solution at 37 °C for visualization of viable myocardial tissue. Processed sections from CpG B injected animals (n = 25) and PBS injected animals (n = 35) were digitally photographed and analyzed for the extension of AaR (the non-Evans blue stained portion of the myocardium) and necrosis (the non-TTC stained portion of the AaR), using Adobe Photoshop (Adobe Systems Incorporated, San Jose, CA, USA). An investigator blinded for previous the intervention performed photograph analysis.

### RNA extraction and PCR analysis

Total RNA from hearts were extracted using TRIzol (Invitrogen, Life Technologies Corporation, CA, USA) and purified using RNeasy Mini Columns (QIAGEN, Hilden, Germany). cDNA was synthesized using High Capacity cDNA Reverse Transcription Kit (Applied Biosystems, Carlsbad, CA, USA). Quantification of gene expression was performed by quantitative real-time PCR, using Power Sybr Green Master Mix (Applied Biosystems) and sequence specific PCR-primers (Table 1). Gene expression of the housekeeping gene glyceraldehyde 3-phosphate dehydrogenase (GAPDH) was used as reference for relative quantifications.

**Table 1:** Primer sequences used in qPCR assays.

Target	Species	Sequence (5′→3′)	Acc.nr
**GAPDH**	Rat/Mouse	(+)-CCAAGGTCATCCATGACAACTT	NM_008084
		(−)-AGGGGCCATCCACAGTCTT	
**CXCL2**	Mouse	(+)-CACCCAAACCGAAGTCATAGC	NM_008176
		(−)-AATTTTCTGAACCAAGGGAGCTT	
**TNFα**	Mouse	(+)-AGACCCTCACACTCAGATCATCTTC	NM_013693
		(−)-CCACTTGGTGGTTTGCTACGA	
**IFNβ**	Mouse	(+)-CCATCATGAACAACAGGTGGAT	NM_010510
		(−)-GAGAGGGCTGTGGTGGAGAA	
**CD14**	Mouse	(+)-TTCAGAATCTACCGACCATGGA	NM_009841
		(−)-GATCTGAGAAGTTGCAGGAACAAC	
**IL-1α**	Mouse	(+)-ACCCATGATCTGGAAGAGACCA	NM_010554
		(−)-CTGACGAGCTTCATCAGTTTGTATC	
**IL-1β**	Mouse	(+)-CTACAGGCTCCGAGATGAACAAC	NM_008361
		(−)-CAAAGCTCATGGAGAATATCACTTGT	
**IL-1RA**	Mouse	(+)-CAAGCTCCAGCTGGAGGAAGT	NM_031167
		(−)-TCTAGTGTTGTGCAGAGGAACCA	
**CCL2**	Mouse	(+)-AAAGAAGCTGTAGTTTTTGTCACCAA	NM_011333
		(−)-TTAATGTATGTCTGGACCCATTCCT	

GAPDH was chosen as housekeeping gene.

### Flow cytometry analysis

Whole blood (100 µl) was incubated with Mouse BD Fc Block (BD Biosciences, San Jose, CA, USA) for 5 minutes at 4 °C, after which samples were added 2.5 µl CD11b-APC (0.2 mg/ml) and Ly6G-PE (0.2 mg/ml) (BD Biosciences), and incubated for 30 minutes in the dark at room temperature. Samples were then added FACS Lysing Solution (BD Biosciences) and incubated for another 10 minutes at room temperature, before subsequent centrifugation (500×*g*/5 minutes). The supernatant was discarded, and cell pellet was washed twice in buffer (phosphate-buffered saline [PBS] containing 0.5% bovine serum albumin [BSA, Sigma Aldrich]) and centrifuged (500×*g*/5 minutes). After the final centrifugation, supernatant was discarded and samples were added 500 µl 1% paraformaldehyde. Flow cytometry analysis was performed using FACSCalibur (BD Biosciences).

### Immunohistochemical analysis

Sections of formalin-fixed and paraffin-embedded whole hearts were treated with 3% H_2_O_2_, followed by high-temperature unmasking in citrate buffer (pH = 6). Blocking was performed using 0.5% BSA (Sigma Aldrich), before 1 hour incubation with primary antibody against murine Ly6G (1∶100; Abcam, Cambridge, UK) and MAC-2 (1∶1500, Cedarlane, Burlington, ON, Canada) at room temperature. Isotype control staining using IgG2b-κ (Biolegend, San Diego, CA, USA) was included to determine non-specific binding. After washing, slides were incubated 30 minutes with peroxidase-conjugated secondary antibody (ImPRESS Anti-Rat Ig, Vector Laboratories, Burlingame, CA, USA) at room temperature. Sections were developed 7 minutes with chromogen for immunoperoxidase staining (DAB, Vector Laboratories), before counterstaining for hematoxylin (Vector Laboratories). All slides were scanned using Mirax Scan (Carl Zeiss, Oberkochen, Germany) and the images were blinded prior to analysis. The whole left ventricle, including septum, was manually evaluated for positive staining, before automated counting using ImageJ.

### Myeloperoxidase activation assay

Heart protein lysate was obtained by granulation of heart tissue in liquid nitrogen and subsequent resuspension in T-PER Tissue Protein Extraction Reagent (Thermo Scientific, Rockford, IL, USA), containing 1× Halt Protease and phosphatase inhibitor cocktail (Thermo Scientific). The suspension was homogenized using Omni TH homogenizer (Omni International, Kennesaw, GA, USA), and homogenate was cleared by centrifugation (13 500×g/10 min at 4 °C). Protein concentration was measured using the Pierce BCA Protein Assay (Thermo Scientific) according to the specifications supplied by the manufacturer. Myeloperoxidase (MPO) activity was analyzed using the EnzChek Myeloperoxidase (MPO) Activity Assay Kit (Invitrogen), according to the instructions by the supplier. In brief, a total of 0.5 µg protein lysate was added 50 µl Amplex UltraRed substrate and incubated 30 minutes at room temperature. Fluorescence was measured after 30 minutes using Synergy H1 hybrid reader (BioTek, Highland Park, Illinois, USA).

### Statistical analysis

Data were analyzed using GraphPad Prism 5 (GraphPad, San Diego, CA, USA). All values are presented as mean ± SEM. Mann-Whitney U test was used for comparison of two groups. Probability values of *P*<0.05 (2-sided) were considered statistically significant.

## Results

### Impact of increased TLR9 activity on infarct extension upon I/R

**Figure 1:**
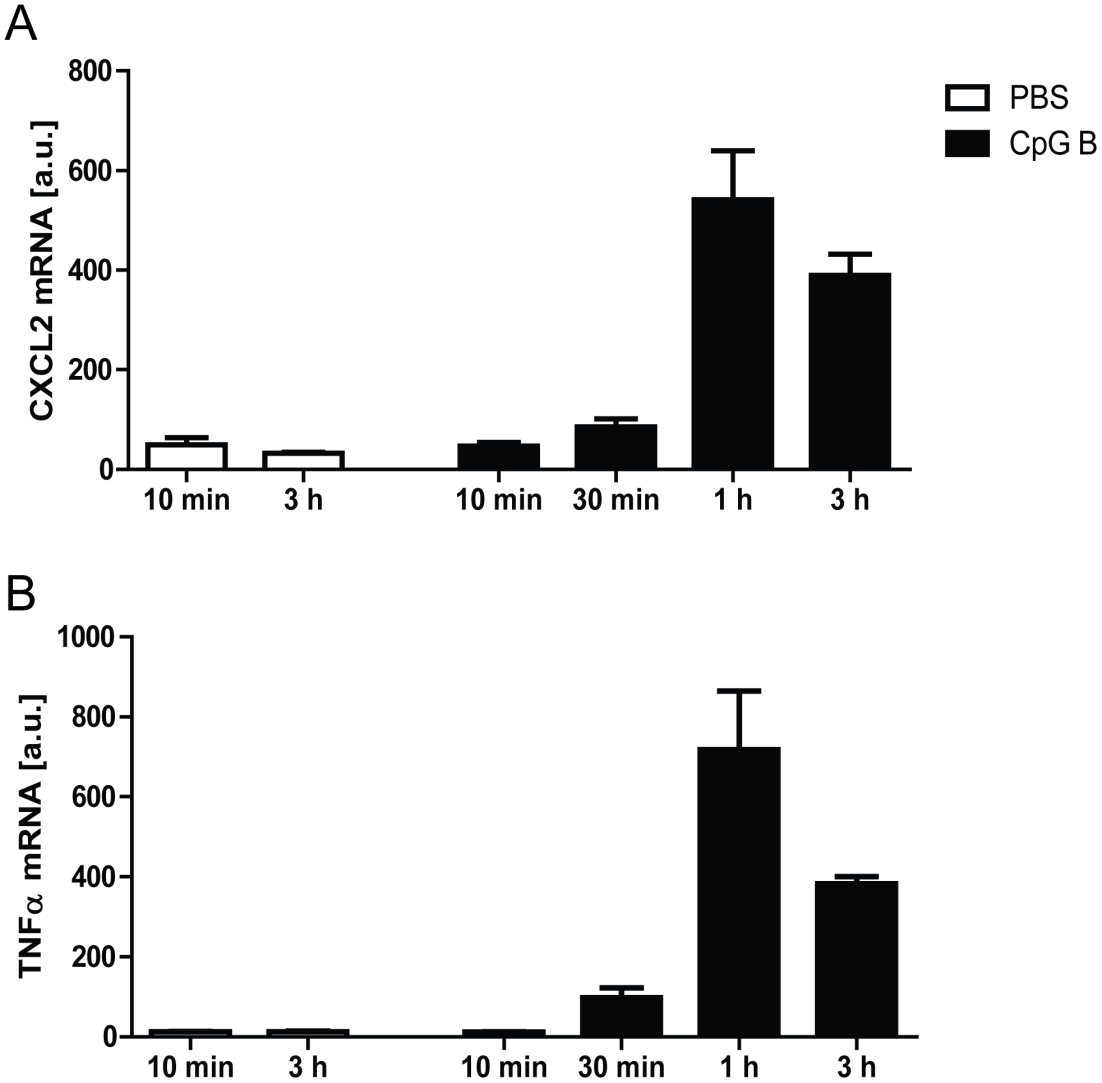
*In vivo* pharmacological assessments of TLR9-activation/inhibition. **Temporal profiles**: Male C57BL/6 mice were injected i.p. with 100 µl 50 µg CpG B (black bars) or vehicle (white bars). Hearts were explanted at different time points (10 min, 30 min, 1 h and 3 h) and TLR9-mediated response was analyzed by mRNA expression levels of CXCL2 (panel A) and TNFα (panel B). Each data point represents mean ± SEM of 5 mice.

**Figure 2:**
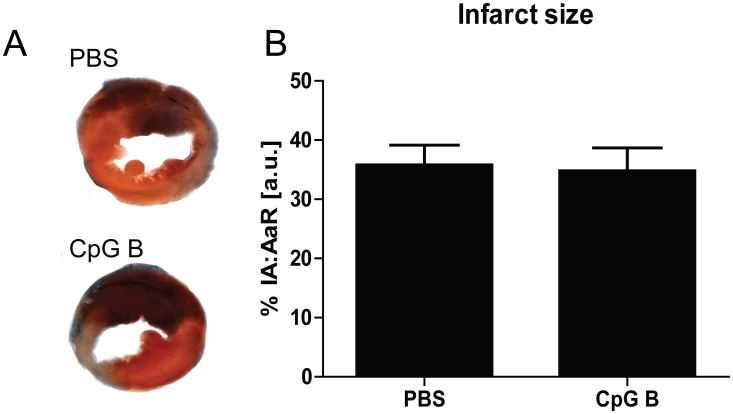
Infarct size. MI was induced in male and female C57BL/6 mice by ligation of LAD, and immediately i.p. injected with CpG B (n = 25) or vehicle (n = 35). Hearts were reperfused after 30 minutes of ischemia. After 24 hours of reperfusion, LAD was re-ligated, area at risk was visualized by coronary perfusion of Evans-blue and viable myocardium was demonstrated by TTC-staining including all animals. Infarct size was calculated as ischemic area (IA) in percent of area at risk (AaR). Panel A: representative photograph of Evans blue and TTC-stained murine heart sections; treated with PBS or CpG B, respectively. Blue color: viable area, red color: AaR, white color: IA. Panel B: Bar-graph representation of infarct extension as calculated from IA and AaR. Each data point represents mean ± SEM.

In total, four deaths were observed during the peri –and –postoperative period. Only two of these deaths were spontaneous (i.e. not due to surgical complication). In order to evaluate the impact of increased TLR9-activities during I/R on subsequent infarct size, mice were immediately injected i.p. with 50 µg of CpG B upon induction of infarction. Cardiac bioavailability of this compound, within a time frame considered pivotal for influencing I/R-induced cardiac injuries, was proven as readily detectable TLR9-stimulated cardiac increases of CXCL2 and TNFα mRNA expression levels could be demonstrated after only 30 minutes (Fig. 1A–B). However, TLR9-stimulation with CpG B did not influence infarct extension (Fig. 2) and this pattern was seen also after sub-grouping into males and females (data not shown).

### Cardiac inflammatory responses upon increased TLR9 activity in I/R

**Figure 3:**
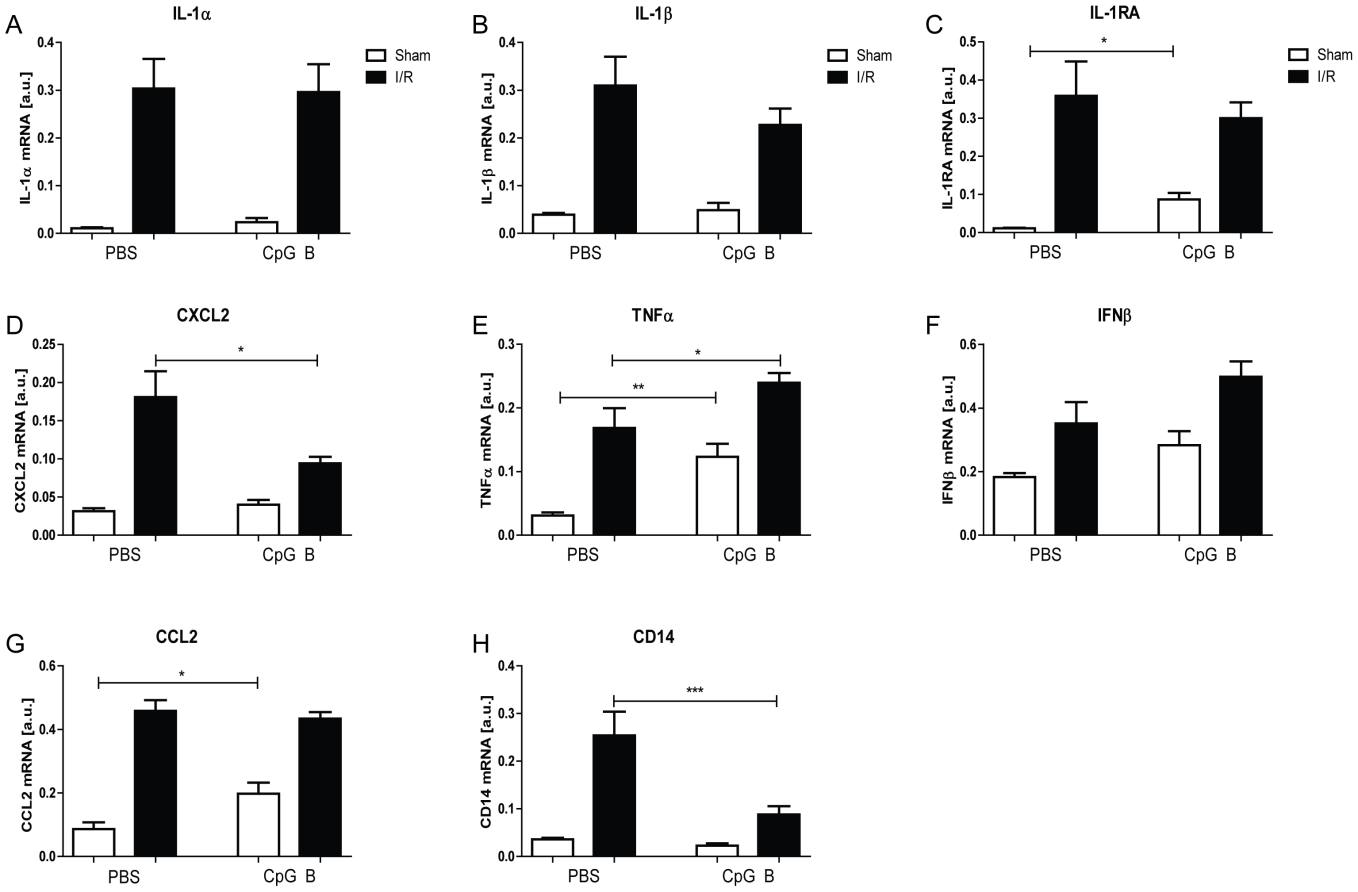
PCR. Male and female C57BL/6 mice were subjected to I/R (black bars) or sham-operation (white bars) with i.p. injections of CpG B (sham: n = 5, I/R: n = 10) or vehicle (sham: n = 5, I/R: n = 10). After 24 hours of reperfusion, hearts were explanted and analyzed by real time RT-PCR for the expression levels of IL-1α, IL-1β, IL-1RA, CXCL1, TNFα, IFNβ, CCL2 and CD14 (Panels A–H). Each data point represents mean ± SEM. *p<0.05 vs. PBS-injection, ** *p<0.01* vs. PBS-injection, ***p<0.001 vs. PBS-injection.

To see if the lack of effect of TLR9 stimulation on infarct size could reflect its inability to modulate myocardial inflammation, we examined mRNA levels of selected chemokines and cytokines in addition to TNFα in both sham-operated and I/R exposed hearts after 24 hours of reperfusion (Figure 3). In sham-operated hearts, CpG B induced a significant increase in interleukin-1 receptor antagonist (IL-1Ra), TNFα and CCL2 compared with sham operated hearts treated with vehicle (Fig. 3C, 3E and 3G). Furthermore, I/R alone induced an up-regulation of all selected genes when compared with sham, and notably, TLR9-stimulation did regulate cardiac expression of some of the examined inflammatory genes beyond that of I/R. Thus, a significant down-regulation of CXCL2 (Fig. 3D) and a significant up-regulation of TNFα (Fig. 3E) were found when comparing CpG B with vehicle-treated I/R mice. In CpG B treated mice, these findings were accompanied by a marked down-regulation of mRNA levels of CD14 as a marker of monocyte infiltration (Fig. 3H).

### Increased TLR9-activity and effect on circulating immune cells in I/R

**Figure 4:**
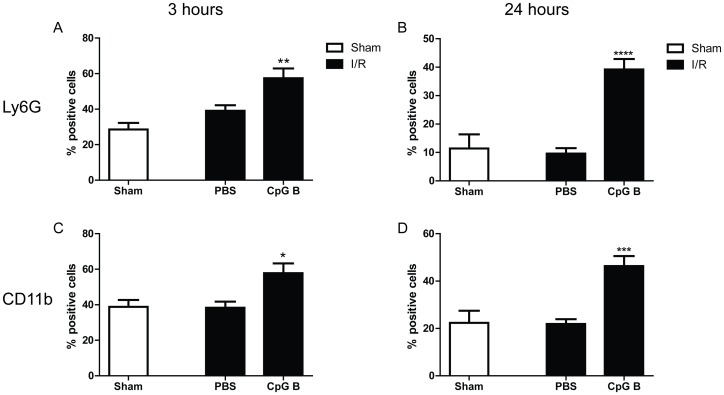
Flow cytometry. Presence of circulating Ly6G^+^ and CD11b^+^ cells were analyzed by flow cytometry of whole blood from male and female C57BL/6 mice injected with CpG B (n = 10) or vehicle (n = 10) upon I/R (black bars), 3 and 24 hours after reperfusion. Sham-operated mice, who received vehicle-injections, were used as controls (n = 5, white bars). Each data point represents mean ± SEM. *p<0.05 vs. PBS-injection, ** *p<0.01* vs. PBS-injection, ***p<0.001 vs. PBS-injection.

We next analyzed relative amounts of monocytes (CD11b^+^) and granulocytes (Ly6G^+^) in peripheral blood, extracted from mice 3 and 24 hours following I/R by flow cytometry (Fig. 4A–D). I/R alone did not affect the concentrations of investigated cell-types (Fig. 4A). However, systemic stimulation of TLR9 mediated a robust and significant increase in proportion of monocytes and granulocytes both at 3 and 24 hours (Fig. 4A–D).

### Effects of increased TLR9-activity on cardiac infiltration of immune cells upon I/R

**Figure 5:**
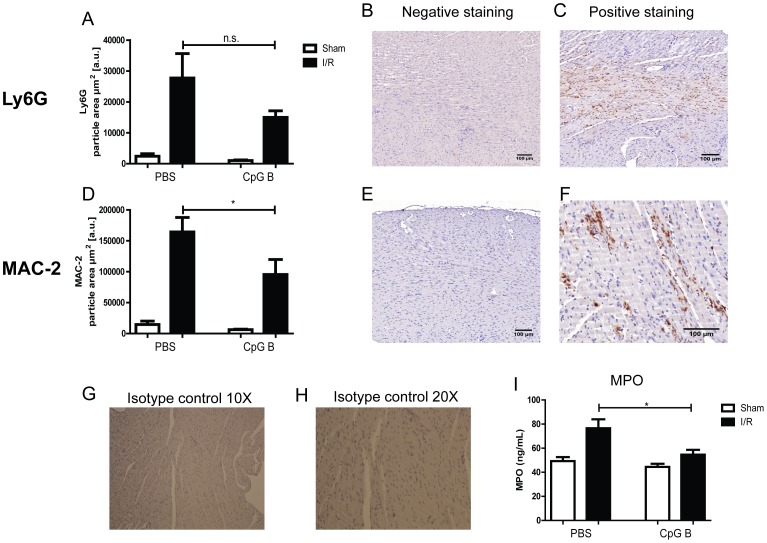
Immunohistochemistry and MPO-activity. Immunohistochemical staining of Ly6G^+^ (panels A–C) and MAC-2^+^ (panels D–F) cells were analyzed in hearts of male and female C57BL/6 mice, subjected to I/R (black bars) or sham-operation (white bars) with i.p. injections of CpG B (sham: n = 6, I/R: n = 12) or vehicle (sham: n = 6, I/R: n = 12). Each data point represents mean ± SEM. *p<0.05 vs. PBS-injection. Panels B–C and E–F depicts representative photographs of negative and positive staining for Ly6G^+^ and MAC-2^+^ cells, respectively. Evaluation of putative non-specific binding was performed using isotype specific IgG (IgG2b-κ) and photographed at 10X (panel G) and 20X (panel H). Protein lysate of hearts from male and female C57BL/6 mice, subjected to I/R (black bars) or sham-operation (white bars) with i.p. injections of CpG B (sham: n = 5, I/R: n = 10) or vehicle (sham: n = 5, I/R: n = 10) were analyzed for myeloperoxidase (MPO) activity (panel I). Each data point represents mean ± SEM. *p<0.05 vs. PBS-injection.

MI leads to cardiac infiltration of various immune cells, and different cellular subgroups typically infiltrate at different time-dependent phases after infarction. In order to quantify the magnitude of these cellular activities, we performed immunohistochemistry on markers for granulocytes (Ly6G^+^) and monocytes/macrophages (MAC-2^+^) 24 hours after I/R. Isotype controls showed no unspecific binding of primary antibodies (Fig. 5G–H). As expected, a substantial cardiac infiltration of granulocytes could be detected upon I/R (Fig. 5), and this was not significantly affected by concomitant systemic TLR9-activation (Fig. 5A–C). However, CpG B led to significantly lower cardiac MPO activity suggesting a down-regulatory effect on granulocyte function (Fig. 5I). However, in line with the effect on CD14 mRNA levels, CpG B induced a marked lower cardiac amount of monocytes/macrophages during I/R (Fig. 5D–F).

## Discussion

The main objective of this study was to investigate the effect of TLR9-activation during onset of myocardial I/R with respect to infarct size. Although TLR9-activation elicited robust increases in systemic inflammation as well as a decrease in some inflammatory markers within the myocardium, these systemic and cardiac inflammatory alterations did not translate into changes in infarct size. Several previous reports have convincingly demonstrated beneficial effects of TLR-stimulation, including TLR9 [5], [12]–[15], initiated *before* ischemia. These studies do advocate for a putative role of TLR-activation in clinical situations of expected cardiac ischemia (for instance coronary artery bypass grafting). However, no studies have investigated the effects of TLR-stimulation restricted to the more common clinical scenario; i.e. intervention *upon* ischemia/reperfusion.

Even though still clinically relevant, our experimental setup does not allow discriminating the effects of our intervention on ischemia, reperfusion or a combination of both. In order to specifically address the reperfusion phase of I/R, drugs should preferably be injected directly into the occluded artery upon reperfusion. As to our knowledge, this is not feasible in a murine I/R-model. However, a requirement for substantial cardiac influences during ischemia necessitates drugs to enter deep into the myocardium at risk (which requires substantial coronary perfusion by non-culprit arteries) as well as temporal accessibility within the time-range of ischemia. As to the latter, we were not able to detect increased TLR9-signaling at earlier time-points than 30 minutes post-injection. Thus, even though we cannot exclude cardiac influences during ischemia, our experimental set-up suggests a predominant effect of TLR9-stimulation during reperfusion.

Our main parameter, i.e. infarct extension at 24 hours, was similar between TLR9 stimulated and vehicle treated I/R mice However, we do provide evidence of substantial systemic inflammatory responses upon i.p. injection of CpG B. After CpG B injection, significant increases of both circulating granulocytes and monocytes could be demonstrated in both sham-operated mice and mice subjected to I/R. However, concomitant with these peripheral cellular increments, we also provide evidence, at least in some degree, of the opposite pattern in the infarcted myocardium. Immunohistochemisty revealed a significant reduction of cardiac cells expressing MAC-2, indicating a lower number of monocytes infiltrating the infarcted myocardium upon systemic TLR9 activation. This observation was further supported by decreased myocardial CD14 expression during I/R upon CpG B activation. Moreover, although we found no alteration in myocardial infiltration of granulocytes in infarcted hearts treated with CpG B, there was a marked decrease in MPO activity suggesting inhibitory effect on the functional capacity of these cells. Thus, while activation of TLR9 seems to increase systemic inflammation, the cardiac responses upon I/R and systemic TLR9-activation were opposite. We do not provide data as to explain these unexpected results. However, the attenuated infiltration of monocytes and granulocytes in the ischemic myocardium upon systemic TLR9-activation may possibly be caused by retention and clustering of these cells within the circulation. As for granulocytes, similar systemic retention has been reported after stimulation with glucocorticosteroids [18]. The down-regulatory effect of TLR9-stimulated myocardial CCL2-expression a chemokine that is strongly expressed within the myocardium [19], [20], could also have contributed to the decreased attraction of leukocytes into the myocardium. These issues will have to be further investigated in forthcoming studies.

Regardless of mechanism, the reduced infiltration of monocytes/macrophages and reduced cardiac MPO activity during I/R upon systemic TLR9 activation did not impact infarct size. Previous studies have demonstrated that altered myocardial infiltration of immune cells in MI can influence the final extent of cardiac injury [21]–[24]. However, an unequivocal conclusion regarding the consequence of altered cardiac immune cell infiltration as a whole is non-existing. Although our findings could provide a foundation for questioning a direct link between myocardial inflammation in general and I/R injury, the effects of systemic TLR9-activation are multifaceted and thus our data must be interpreted with caution. Also, although we found some anti-inflammatory effects of CpG B stimulation within the myocardium, we demonstrated enhanced TNFα expression, which could have counteracted the potential beneficial anti-inflammatory effects of TLR9 activation. Moreover, TLR9 activation promoted systemically altered cellular and inflammatory responses, and these systemic responses could have putative indirect consequences on the heart. In such, our interventional approach is complex and we cannot exclude the possibility of a different outcome if we had means of isolating the TLR9 stimulatory effects to the heart. Also, we do emphasize that our conclusions are restricted to the evaluation of infarct extension at 24 hours. That said, we provide no data as to the consequences of systemic TLR9 activation on myocardial function, nor putative TLR9-stimulated regulation on subsequent infarct and cardiac remodeling during the time-period beyond 24 hours.

In conclusion, systemic TLR9-activation upon onset of ischemia results in definite alterations in cardiac, as well as systemic, inflammatory responses, but does not impact infarct extension. This study questions the role for pharmacological targeting of the TLR9-system during cardiac I/R.
